# How accurate are prenatal tractography results? A postnatal in vivo follow-up study using diffusion tensor imaging

**DOI:** 10.1007/s00247-017-3982-y

**Published:** 2018-03-17

**Authors:** Jae W. Song, Gerlinde M. Gruber, Janina M. Patsch, Rainer Seidl, Daniela Prayer, Gregor Kasprian

**Affiliations:** 1 Department of Radiology, Massachusetts General Hospital,, Harvard Medical School,, Boston, MA USA; 2 Center for Anatomy and Cell Biology, Department of Systematic Anatomy,, Medical University of Vienna,, Vienna, Austria; 3 Department of Biomedical Imaging and Image-guided Therapy,, Medical University of Vienna,, Währinger Gürtel 18-20, 1090 Vienna, Austria; 4 Department of Pediatrics and Adolescent Medicine,, Medical University of Vienna,, Vienna, Austria

**Keywords:** Brain, Corpus callosum, Corticospinal tract, Diffusion tensor imaging, Fetus, Infant, Magnetic resonance imaging, Tractography

## Abstract

Prenatal detection of abnormal white matter tracts might serve as a structural marker for altered neurodevelopment. As a result of many technical and patient-related challenges, the accuracy of prenatal tractography remains unknown. We hypothesized that characteristics of prenatal tractography of the corpus callosum and corticospinal tracts derived from fetal diffusion tensor imaging (DTI) data are accurate and predictive of the integrity of these tracts postnatally. We compared callosal and corticospinal tracts of 12 subjects with paired prenatal (age: 23–35 gestational weeks) and postnatal (age: 1 day to 2 years) DTI examinations (b values of 0 s/mm^2^ and 700 s/mm^2^, 16 gradient encoding directions) using deterministic tractography. Evaluation for the presence of callosal segments and corticospinal tracts showed moderate degrees of accuracy (67–75%) for the four segments of the corpus callosum and moderate to high degrees of accuracy (75–92%) for the corticospinal tracts. Positive predictive values for segments of the corpus callosum ranged from 50% to 100% and for the corticospinal tracts, 89% to 100%. Negative predictive values for segments of the corpus callosum ranged from 25% to 80% and for the corticospinal tracts, 33% to 50%. The results suggest that when the tracts are not well characterized on the fetal MR, predictions about the postnatal tracts are difficult to make. However, accounting for brain maturation, prenatal visualization of the main projection and commissural tracts can be clinically used as an important predictive tool in the context of image interpretation for the assessment of fetal brain malformations.

## Introduction

The number of our brain network connections determines our neurological and cognitive abilities [1, 2]. MRI allows for noninvasive examinations of these connections on a structural and functional level. In cases of clinical deficits, MRI further provides noninvasive insights into pathological changes of brain connectivity. Thus MRI is particularly relevant in the field of prenatal imaging [3] as physicians are asked to predict the functional consequences of structural brain abnormalities. As a promising approach to optimizing prenatal counseling in cases of fetal brain abnormalities, diffusion tensor imaging (DTI) has recently revolutionized our ability to visualize the structural connectivity of prenatal brains [4–9].

As a result of fetal motion, acquiring DTI prenatally is challenging. However, short acquisition times and large voxel sizes permit reconstruction of the main projection, commissural and occasionally also association fiber pathways using deterministic tractography. Assessing the integrity of these fiber tracts in the setting of a fetal brain abnormality might become an important clinical tool to identify future neurological deficits and facilitate parental counseling. Moreover, DTI characterizes severely deranged white matter pathway architecture in complex fetal brain malformations, thereby permitting comprehensive morphological descriptions and radiologic diagnoses [7]. Currently, the anatomical validity of the visualized pathways in fetal DTI is increasingly recognized in postmortem correlation studies [5]. However the accuracy and predictive value of prenatal tractography results for postnatal tractography remain unknown.

Given technical and patient-related challenges to performing fetal MR, the accuracy and predictability of white matter tractography in fetal MR has not been systematically investigated. The researchers in this study aimed to understand the relevance and accuracy of fetal tractography in predicting the integrity of postnatal pathways. We hypothesized that characteristics of prenatal tractography of the corpus callosum and corticospinal tracts derived from fetal DTI data are accurate and predictive of the integrity of these tracts postnatally.

## Materials and methods

### Subjects

This study is a retrospective observational study conducted between April 2006 and July 2015. The inclusion criteria for this study were the presence of both prenatal and postnatal MRIs, performed at the Department of Biomedical Imaging and Image-guided Therapy of the Medical University of Vienna, Austria, and no interventions to the brain in the interval of the two MRI examinations. We identified 75 subjects with both fetal and postnatal MRIs. We checked each examination for DTI sequence acquisitions. Fourteen fetal MRs and 32 postnatal MRs resulted in 13 subjects with both fetal and postnatal MRs with DTI sequences. After assessing the 13 prenatal DTI datasets, we excluded one case because of poor signal to noise. Thus we included 12 cases with paired fetal and postnatal MRI with DTI.

The 12 subjects comprised 7 girls and 5 boys. Gestational age was determined by biometry during the first sonographic examination and is presented as postconceptional age. The mean gestational weeks of age of the fetuses was 30 and 1/7 weeks (range: 23 and 3/7 weeks to 35 and 2/7 weeks). The mean postnatal age was 5 months (range: 1 day to 2 years). The mean interval time between the fetal and postnatal DTI exams was 262 days (range: 12–1,081 days). Eight cases had primary central nervous system (CNS) pathologies, three had secondary CNS pathologies and one had a non-neurological congenital diagnosis (Table 1). The categorization of primary versus secondary CNS pathologies was based on whether the pathology was derived from a neurologic origin or was a result of a more systemic pathology. For instance, pathologies defined as primary CNS pathology included lissencephaly or septo-optic dysplasia. A secondary CNS pathology included middle cerebellar artery vascular infarct in the context of congenital heart disease (tetralogy of Fallot). Only one case was thought to be in the “other” category, with no known obvious CNS pathology; that case was imaged for the clinical condition of gastroschisis.

**Table 1:** Demographics

Case	Gender	Age (gestational weeks+days) at prenatal imaging	Age at postnatal imaging	MR scanner	Category of pathology	Type
1	M	34+2	8 days	Philips Intera	Other	Gastroschisis
2	M	24+2	2 years	Philips Intera	Primary	Periventricular leukomalacia, germinal matrix cysts, cortical dysplasia
3	M	23+3	2 years	Philips Intera	Primary	Lissencephaly
4	F	30+5	7 months	Philips Intera	Primary	Lissencephaly, aqueductal stenosis with hydrocephalus
5	F	27+2	3 months	Philips Intera	Primary	Hindbrain malformation
6	M	33+6	1 day	Philips Intera	Primary	Cerebellar capillary hemangioma complicated by hemorrhage
7	F	32+4	5 days	Siemens Aera	Primary	Unilateral left ventriculomegaly of unknown etiology
8	M	26+6	4 days	Philips Intera	Secondary	Tetralogy of Fallot complicated by left MCA infarct
9	M	29+4	5 days	Siemens Aera	Primary	Parieto-occipital cyst
10	F	28+2	7 months	Siemens Aera	Secondary	Cortical infarcts from intrauterine fetal demise twin gestation
11	F	35+2	2 days	Philips Intera	Primary	Septo-optic dysplasia, aqueductal stenosis with hydrocephalus
12	M	33+6	4 months	Philips Intera	Secondary	In utero MCA infarct with right-side ventriculomegaly

*F* female, *M* male, *MCA* middle cerebral artery

Data were obtained from pregnant women who were informed about the MR examination and possible risks. The ethics committee of the Medical University of Vienna approved the protocol and waived written informed consent because of the study’s retrospective design. We anonymized all image data by case number before further analysis.

### Imaging technique and segmentation

Prenatal imaging was performed on a 1.5-T MR (Gyroscan Intera; Philips Healthcare, Best, the Netherlands) and a five-element phased-array channel cardiac coil. In addition to the standard structural fetal MR sequences (multiplanar T2-W single shot turbo spin echo [TSE], echo time [TE] 140 ms, repetition time [TR] shortest, field of view [FOV] 230–260 mm, flip angle 90°, matrix 256x153, voxel size 0.78/1.18 /4.00 mm; multiplanar steady-state free precession [SSFP], FOV 260 mm, matrix 192x219, voxel size 1.35/1.19/3 mm, flip angle 80°), a fetal DTI sequence was acquired. The axial single-shot fat-suppressed echoplanar DTI sequence included 16 noncollinear diffusion gradient-encoding directions with b values of 0 s/mm^2^ and 700 s/mm^2^, TE 90 ms, TR shortest, flip angle 90°, matrix 112x105, slice thickness 3 mm and acquisition time of 1 min 16 s. The voxel size was 2.8–4.3 mm^3^. No sedation or intravenous contrast agents were administered. Postnatal imaging was performed on either the same 1.5-T scanner as the prenatal examination (Intera; Philips Healthcare, Best, the Netherlands; TR/TE 4,371/90 ms, b values of 0 s/mm^2^ and 700 s/mm^2^, 16 gradient encoding directions, slice thickness 4 mm, matrix 128x128, acquisition time 4–5 min) or on a Magnetom Aera 1.5 T (Siemens Healthcare, Erlangen, Germany; TR/TE 9,600/120 ms, b values of 0 s/mm^2^ and 700 s/mm^2^, 16 gradient encoding directions, matrix 1,072x1,072, slice thickness 2–3 mm, acquisition time 5–6 min). The voxel size was 0.83 mm^3^. An 8-channel head coil was used for postnatal imaging. All postnatal examinations were performed under sedation and in the presence of an anesthesiologist with dedicated experience in pediatric neuro MRI.

A multiple regions-of-interest (ROI) approach was used to reconstruct the corticospinal tracts and corpus callosum using anatomical knowledge of the tract trajectories as previously described in published protocols [10, 11]. The corticospinal tracts and corpus callosum were segmented using the Philips Intellispace Portal system by two radiologists (J.W.S., with 1 year of experience, and G.K., with 3 years of experience) who were blinded to either prenatal or postnatal tractography to ensure no bias in identifying tract morphology at the different ages.

### Analyses

We performed qualitative analyses including evaluating the presence of each tract at both stages. We evaluated presence as a dichotomous variable — present or not present — by assessing for the presence of fibers accounting for the degree of signal to noise. We also evaluated the corpus callosum by its anatomical parts: rostrum, genu, body and splenium. The rostrum was identified as a beak-shaped segment curving posteriorly from the genu. The genu was identified as the curved anterior portion of the corpus callosum projecting anteriorly to approximately the region of the line connecting the mammillary body, anterior commissure and corpus callosum [12]. The body of the corpus callosum was identified as a horizontal structure in the expected location of the corpus callosum on midline sagittal images. Finally, the splenium was identified as a caudally oriented or bulbous posterior portion [13].

We categorized the morphology and integrity of the corticospinal tracts into the following four categories: (1) not visualized, (2) discontinuous, (3) intact and (4) artifact. We performed this categorization visually by assessing the presence and morphology of the fibers accounting for the degree of signal to noise.

Next we evaluated the utility of fetal DTI as a diagnostic test for assessing corpus callosum and corticospinal tract pathways by tractography. We defined true positives as cases where pathways were identified in both fetal and postnatal DTI by tractography. True negatives were cases where pathways were not identified on both the fetal and postnatal DTI by tractography. False positives were cases where pathways were visualized by fetal tractography but were not present on postnatal tractography. Finally, false negatives were cases where pathways were not visualized on fetal tractography but were present on postnatal tractography. Accuracy was calculated as the total number of true positives and true negatives divided by the sum of true positives, true negatives, false positives and false negatives. We calculated positive predictive values (PPV) as the number of true positives divided by the total number of true positives and false positives. Negative predictive values (NPV) were the number of false positives divided by the total number of true positives and false positives.

We also compared quantitative outcomes including fractional anisotropy (FA) and apparent diffusion coefficient (ADC). We measured the ventricular sizes to evaluate for the degree of ventriculomegaly. The diameter of the atria of the lateral ventricles was measured on coronal T2-weighted images [14]. Ventriculomegaly was defined as an atrial diameter exceeding 10 mm.

## Results

### Corpus callosum

**Fig. 1:**
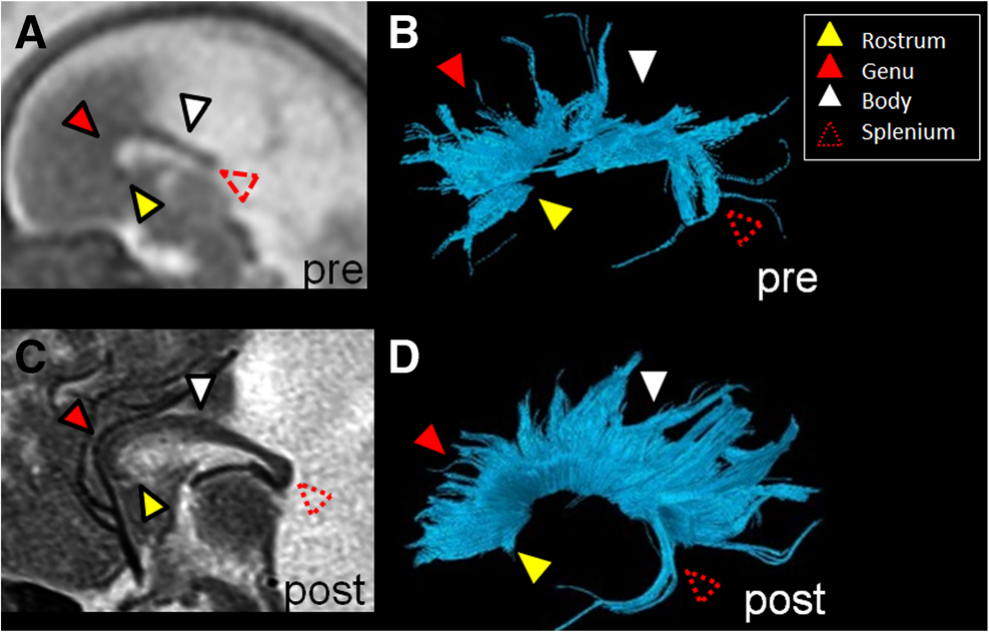
Prenatal and postnatal tractography of the corpus callosum segments in a boy, case 9. **a, b** Prenatal (29+4 gestational weeks) and (**c, d**) postnatal (5 days) sagittal T2-weighted MR images and their tractography pair are illustrated with all four segments of the corpus callosum (*yellow arrowhead* rostrum, *solid red arrowhead* genu, *white arrowhead* body, *dotted red arrowhead* splenium). The prenatal tractography is considered accurate for this case because each of the same segments is identified in the postnatal tractography

**Fig. 2:**
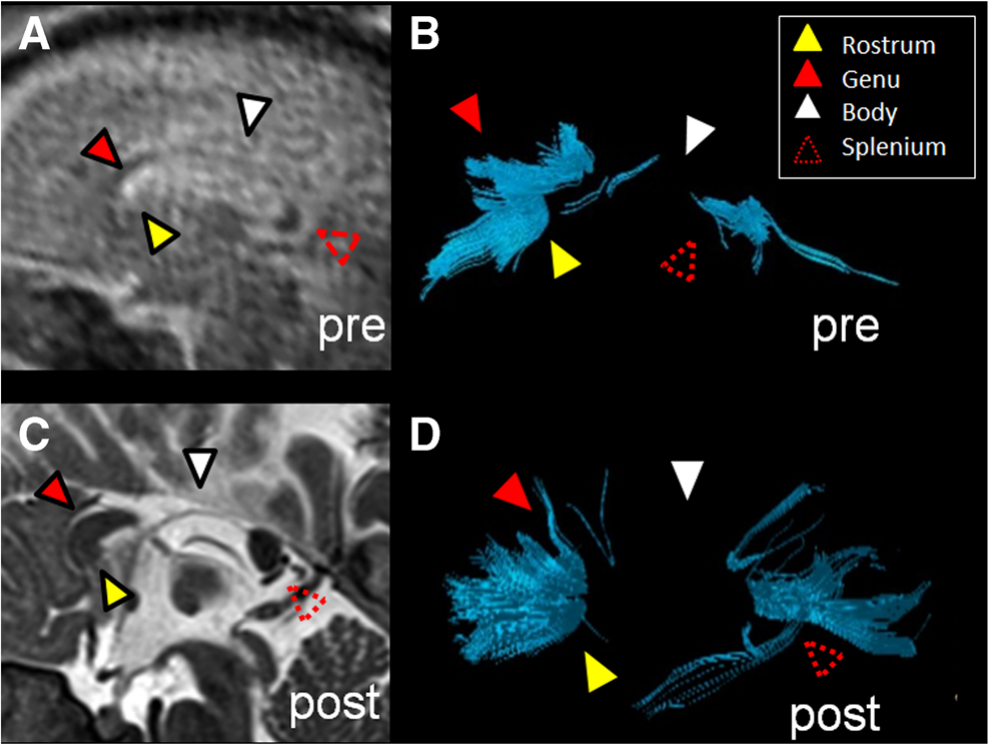
Prenatal and postnatal tractography of the corpus callosum segments in a girl, case 10. **a, b** Prenatal (28+2 gestational weeks) and (**c, d**) postnatal (7 months) tractography pair and their respective sagittal T2-weighted images are illustrated, with only three of the four segments of the corpus callosum (*yellow arrowhead* rostrum, *solid red arrowhead* genu, *white arrowhead* body, *dotted red arrowhead* splenium) identified in the prenatal pair. In the postnatal segmentation of the corpus callosum (**c**, **d**), the same three segments are identified. The prenatal tractography is considered accurate for this case because each of the same three segments is identified in the postnatal tractography. Moreover, as the sagittal T2-weighted postnatal image shows, the body of the corpus callosum is indeed absent

Anatomical segments (rostrum, genu, body and splenium) of the corpus callosum were evaluated as being present or not present by tractography (Figs. 1 and 2, Table 2). Among the callosal segments, the body of the corpus callosum was visualized the least frequently by tractography; it was only visualized 17% (*n*=2 of 12) of the time prenatally and 25% (*n*=3 of 12) of the time postnatally. The rostrum was visualized 42% (*n*=5 of 12) of the time prenatally and 75% (*n*=9 of 12) of the time postnatally. The genu was visualized 67% (*n*=8 of 12) of the time prenatally and 92% (*n*=11 of 12) postnatally. Finally, the splenium was visualized 58% (*n*=7 of 12) of the time prenatally and 75% (*n*=9 of 12) postnatally.

**Table 2:** Corpus callosum tractography reliability analysis

Case	Rostrum		Genu		Body		Splenium	
	Prenatal	Postnatal	Prenatal	Postnatal	Prenatal	Postnatal	Prenatal	Postnatal
1	NP	NP	P	P	NP	NP	P	P
2	P	P	P	P	NP	P	P	P
3	NP	P	P	P	NP	P	P	P
4	NP	P	NP	P	NP	NP	NP	NP
5	P	P	P	P	NP	NP	NP	P
6	NP	P	NP	P	NP	NP	NP	P
7	NP	NP	NP	NP	NP	NP	NP	NP
8	P	P	P	P	P	NP	P	P
9	P	P	P	P	P	P	P	P
10	NP	P	P	P	NP	NP	P	P
11	NP	NP	NP	P	NP	NP	P	NP
12	P	P	P	P	NP	NP	NP	P
	*n*=5	*n*=9	*n*=8	*n*=11	*n*=2	*n*=3	*n*=7	*n*=9

*NP* not present, *P* present

We also assessed the presence of the corpus callosum by segments on midline sagittal T2-weighted images of the brain on fetal and postnatal MR exams and compared it to the presence or absence of the segmented tractography by DTI. There was a moderate to strong correlation between the identifiable segments by MR and the tractography. Table 3 shows the results of the MR evaluation with respect to the tractography findings; for instance, when the rostrum was visible on the MR exam, it was also evaluated by tractography and was categorized as a “matched” result if seen on both anatomical MR and DTI tractography exams. On the fetal MR exams, the tractography results matched the anatomical MR exam 75% (*n*=9 of 12) of the time for the rostrum, 83% (*n*=10 of 12) of the time for the genu, 58% (*n*=7 of 12) of the time for the body and 75% (*n*=9 of 12) of the time for the splenium. On the postnatal MR exams, the tractography results matched the MR imaging findings 83% (*n*=10 of 12) of the time for the rostrum, 100% (*n*=12 of 12) of the time for the genu, 50% (*n*=6 of 12) of the time for the body, and 83% (*n*=10 of 12) of the time for the splenium.

**Table 3:** Correlation of tractography with MR visualization of the corpus callosum segments

Case	Prenatal				Postnatal			
	Rostrum	Genu	Body	Splenium	Rostrum	Genu	Body	Splenium
1	NM	M	NM	M	NM	M	NM	M
2	NM	M	NM	NM	M	M	M	M
3	M	NM	NM	M	M	M	M	M
4	M	M	M	M	M	M	NM	NM
5	M	M	NM	NM	M	M	NM	NM
6	M	M	M	M	M	M	NM	M
7	M	NM	M	M	NM	M	M	M
8	M	M	M	M	M	M	NM	M
9	M	M	M	M	M	M	M	M
10	NM	M	M	M	M	M	M	M
11	M	M	M	M	M	M	M	M
12	M	M	NM	NM	M	M	NM	M

*M* matched, *NM* not matched

Accuracy values for the genu and body were 75% (*n*=9 of 12), for the rostrum 67% (*n*=8 of 12), and for the splenium 67% (*n*=8 of 12; Table 2). These results suggest that the presence or absence of segments of the corpus callosum by tractography on fetal DTI is moderately accurate for the presence of these fiber pathways by postnatal DTI imaging, even in the setting of underlying neuropathology. There were a 100% PPV for the rostrum and genu, 50% PPV for the body and an 87.5% PPV for the splenium (Table 4). Negative predictive values ranged from 25% to 80%, as shown in Table 4. These findings suggest a very high degree of PPV for prenatal tractography of the corpus callosum.

**Table 4:** Positive and negative predictive values of fetal diffuse tensor imaging (DTI) in predicting the presence of pathways on postnatal DTI of the corpus callosum

	Corpus callosum				Corticospinal tracts	
	Rostrum	Genu	Body	Splenium	Right	Left
Positive predictive value	100.0%	100.0%	50.0%	87.5%	100.0%	88.9%
Negative predictive value	33.3%	25%	80.0%	50.0%	50.0%	33.3%
Accuracy	66.7%	75.0%	75.0%	66.7%	91.7%	75.0%

**Fig. 3:**
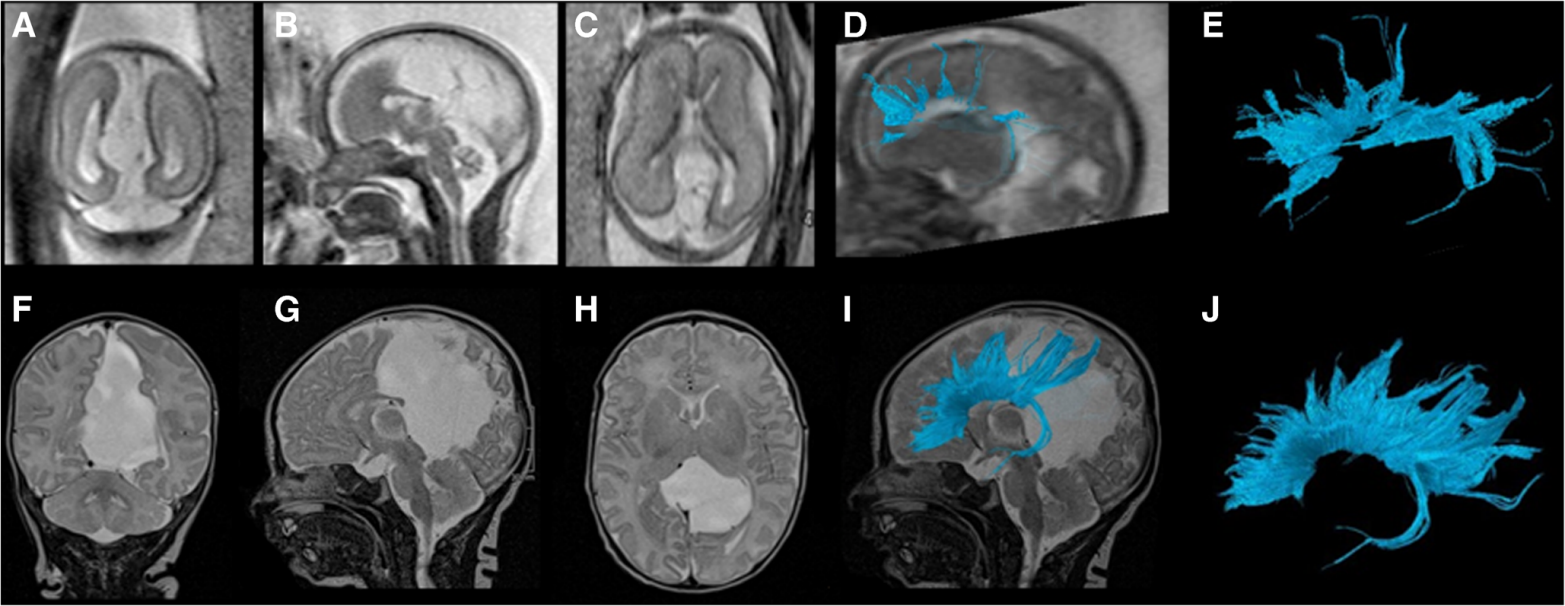
Fetal and postnatal corpus callosum segmentation in a boy (case 9) with a midline parieto-occipital cyst. **a–e** Gestational weeks 29+4. **a** Coronal T2-weighted (TR/TE 10,658/140 ms, flip angle 90°) MR image of the brain. **b, c** Sagittal (**b**) and axial (**c**) T2-weighted MR images of the brain with a midline posterior parieto-occipital cyst. **d** Overlay of the fiber tracts of the corpus callosum on a sagittal T2-weighted image. **e** The rostrum, genu, body and splenium are identifiable on this sagittal diffusion tensor image (DTI) of the corpus callosum in the fetal brain. **f–j** Age 5 days. **f** Coronal T2-weighted (TR/TE 3,070/134 ms, flip angle 180°) MR image of the brain. **g** Sagittal T2-weighted image. **h** Axial T2-weighted image of the brain with a midline posterior parieto-occipital cyst. **i** Segmented sagittal tractography of the corpus callosum. **j** The rostrum, genu, body and splenium are identifiable on sagittal DTI of the corpus callosum in the 5-day-old postnatal brain. *TE* echo time, *TR* repetition time

Figure 3 illustrates a case with a large midline parieto-occipital cyst exerting mass effect on the corpus callosum and thereby displacing the fibers inferiorly and deforming it. Fetal tractography was deemed accurate for all four segments of the postnatal segmentation. For example, in the fetal and postnatal segmentations, the rostrum, genu, body and splenium were all visible by tractography. Consequently, this favored excluding callosal dysgenesis or partial callosal agenesis as a descriptive diagnosis prenatally and impacted counseling.

**Fig. 4:**
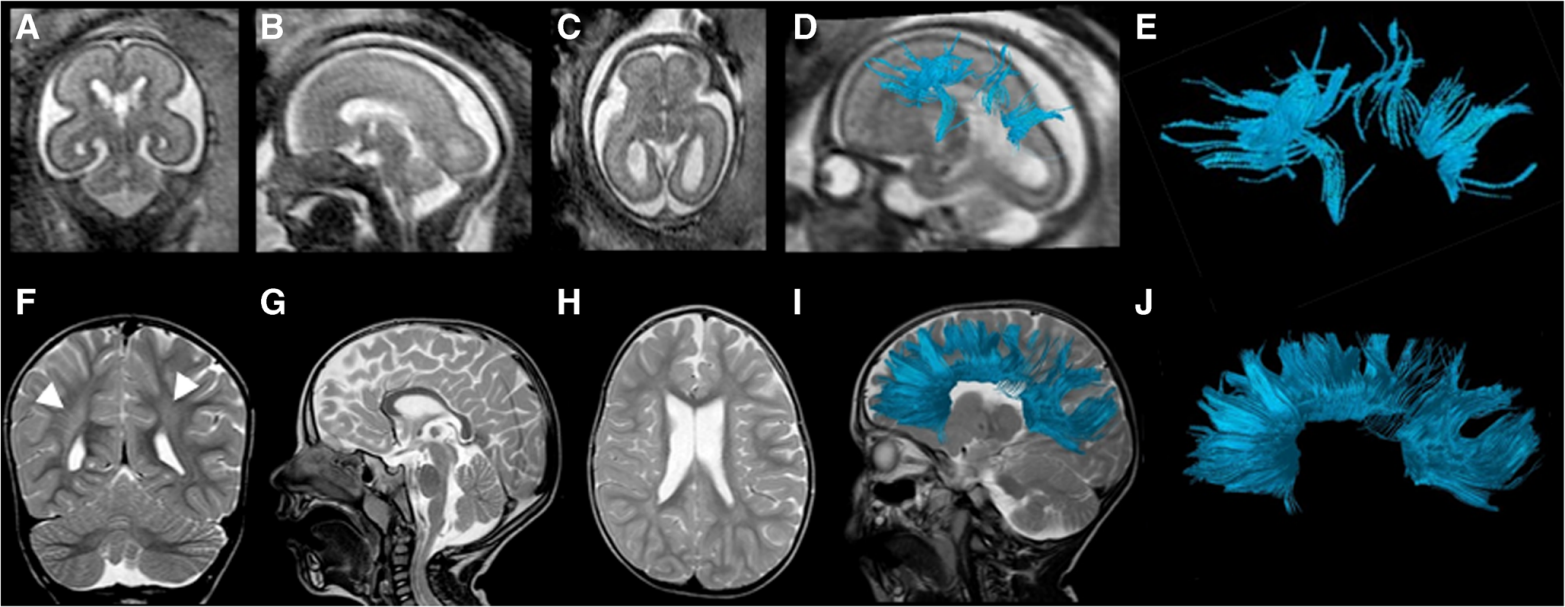
Fetal and postnatal corpus callosum segmentation in a boy (case 2) with periventricular leukomalacia and cortical dysplasia. **a–e** Age 24+2 gestational weeks. **a** Coronal T2-weighted (TR/TE 10,653/140 ms, flip angle 90°) MR image. **b** Sagittal T2-weighted image. **c** Axial T2-weighted image of the fetal brain. **d** Overlay of the fiber tracts of the corpus callosum on a sagittal T2-weighted image. **e** The rostrum and body of the corpus callosum are not well visualized, while the genu and splenium are identifiable on sagittal diffusion tensor imaging (DTI) of the corpus callosum in the fetal brain. **f–j** Age 2 years. **f** Coronal T2-weighted (TR/TE 4,000/90 ms, flip angle 90°) MR image of the brain shows asymmetrical, right greater than left, periventricular T2 prolongation (*arrowheads*), compatible with white matter injury. **g** Sagittal T2-weighted image of the postnatal brain shows an intact corpus callosum. **h** Axial T2-weighted image. **i** Segmented sagittal view of the callosal fiber tracts overlaid on a sagittal T2-weighted image of the postnatal brain appears fully intact. **j** The rostrum, genu, body and splenium are identifiable on this sagittal DTI of the corpus callosum segmentation. *TE* echo time, *TR* repetition time

Figure 4 illustrates a case of periventricular leukomalacia as manifested by subtle periventricular T2 prolongation. At the fetal tractography, the rostrum and body were not fully present. Conversely, in the 2-year-old postnatal DTI all segments of the corpus callosum were present by tractography. In this example, only the genu and splenium were accurate.

**Fig. 5:**
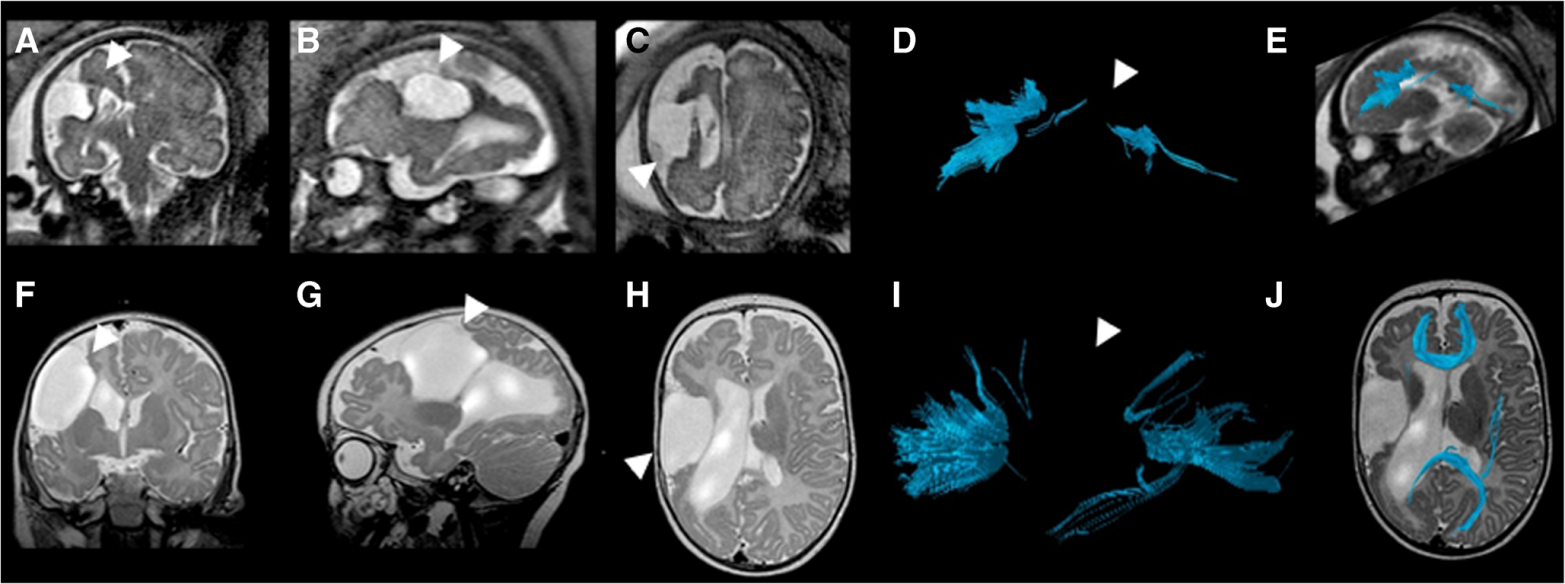
Fetal and postnatal corpus callosum segmentation in a boy (case 12) with right middle cerebral artery territory infarct. **a–e** Age 33+6 gestational weeks. **a** Coronal T2-weighted (TR/TE 2,042/140 ms, flip angle 90°) MR image of the brain reveals cystic encephalomalacia in the region of the right middle cerebral artery infarct (*arrowhead*). **b** Sagittal T2-weighted image of the fetal brain shows infarct (*arrowhead*). **c** Axial T2-weighted image of the fetal brain with infarct (*arrowhead*). **d** Sagittal view of the fiber tract segmentation reveals that the body of the corpus callosum is not visualized in the fetal diffusion tensor imaging (DTI; *arrowhead*). **e** Overlay of the callosal fiber tract on a sagittal T2-weighted image. **f–j** Age 4 months. **f** Coronal T2-weighted (TR/TE 3,000/120 ms, flip angle 90°) MR image of the brain shows sequelae of infarct (*arrowhead*). **g** Sagittal T2-weighted image shows infarct (*arrowhead*). **h** Axial T2-weighted image of the postnatal brain shows infarct (*arrowhead*). **i** Segmented sagittal DTI of the corpus callosum in the postnatal brain reveals that, similar to the fetal DTI, the body of the corpus callosum is not visualized. **j** An overlay of the segmented callosal fibers on an axial T2-weighted image reveals the markedly thinned parenchyma in the region of the right middle cerebral artery territory with cystic encephalomalacic changes. *TE* echo time, *TR* repetition time

Figure 5 illustrates a case of a right middle cerebral artery infarct that resulted in cystic encephalomalacia of the right middle cerebral artery vascular territory. Figure 5 shows a paucity of fibers in the body and splenium of the corpus callosum by fetal tractography. In the 4-month-old postnatal brain, the body of the corpus callosum is also not visualized by tractography. Thus the absence of the body of the corpus callosum by fetal tractography was accurate because it was not seen on postnatal tractography either. However the absence of the splenium in the fetal tractography was not accurate because it was present at postnatal tractography.

We performed a subgroup analysis comparing whether a primary neurologic abnormality (e.g., case 3, lissencephaly) versus secondary (e.g., case 10, cortical infarcts related to intrauterine demise of fetal twin) or unrelated (e.g., case 1, gastroschisis) pathology resulted in less accurate results. Among cases with primary neurologic pathology 50% (*n*=4 of 8) revealed three or more accurate results for the four segments of the corpus callosum. The mean PPV was 100% accounting for all four segments. The mean NPV was 50%. In cases with secondary or unrelated neurologic pathology, 100% (*n*=4 of 4) revealed three or more accurate results between fetal and postnatal segmentations of the corpus callosum. The mean PPV was 92%. No mean NPV was calculated because no true-negative cases for the genu and splenium were present.

We also performed a second subgroup analysis, by fetal age (younger or older than 28 gestational weeks). Four cases comprised fetuses younger than 28 gestational weeks, and eight cases comprised fetuses older than 28 gestational weeks. Among the fetuses younger than 28 gestational weeks, 75% (*n*=3 of 4) of the cases revealed three or more accurate results for the four segments of the corpus callosum. The mean PPV was 70% accounting for all four segments. A mean NPV was not calculated because no true-negative cases for the rostrum, genu or splenium were present. In fetuses older than 28 gestational weeks, 63% (*n*=5 of 8) of the cases showed three or more accurate results among the four segments of the corpus callosum. The mean PPV was 95% accounting for all four segments. A mean NPV was 58%.

**Fig. 6:**
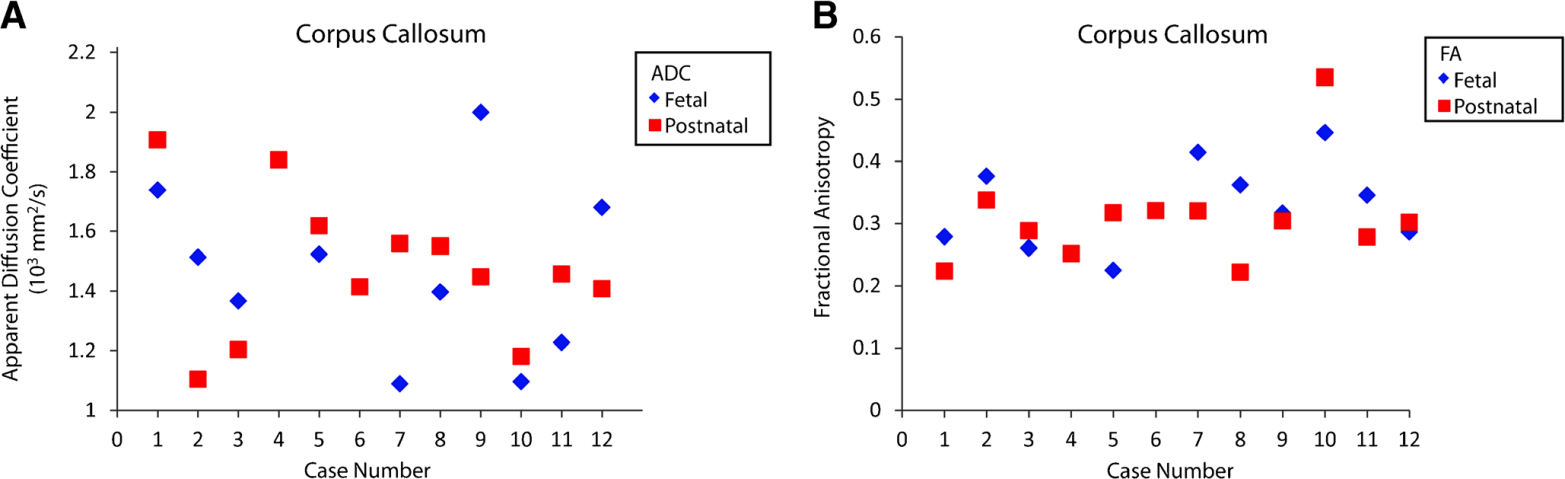
Fetal and postnatal corpus callosum apparent diffusion coefficient (ADC) and fractional anisotropy (FA). **a** Plot shows fetal and postnatal apparent diffusion coefficient for the corpus callosum by case. **b** Plot shows fetal and postnatal fractional anisotropy for the corpus callosum by case. Prenatal outcomes for ADC and FA were not obtainable for cases 4 and 6 because the corpus callosum was not present in these cases

Apparent diffusion coefficient and FA were graphed by paired cases. Visually, the cases demonstrated similar fetal and postnatal outcomes for ADC and FA (Fig. 6).

### Corticospinal tracts

The right and left corticospinal tracts were segmented separately. The PPV was 100% for the right corticospinal tract and 88.9% for the left corticospinal tract. The NPV was 50% for the right and 33% for the left corticospinal tracts. Accuracy values ranged up to 92% (*n*=11 of 12) for the right and 75% (*n*=9 of 12) for the left corticospinal tracts (Tables 4 and 5).

**Table 5:** Corticospinal tract tractography reliability analysis

Case	Right corticospinal tract		Left corticospinal tract	
	Prenatal	Postnatal	Prenatal	Postnatal
1	3	3	3	3
2	3	3	2,4	3
3	3	3	3	3
4	2, 4	3	3	3
5	3	3	3	3
6	3	3	3	3
7	2, 4	2	2, 4	2
8	3	3	3	3
9	3	3	3	3
10	3	3	2,4	3
11	3	3	3	2
12	1	1	3	3

*1* not visualized, *2* discontinuous, *3* intact, *4* artifact

**Fig. 7:**
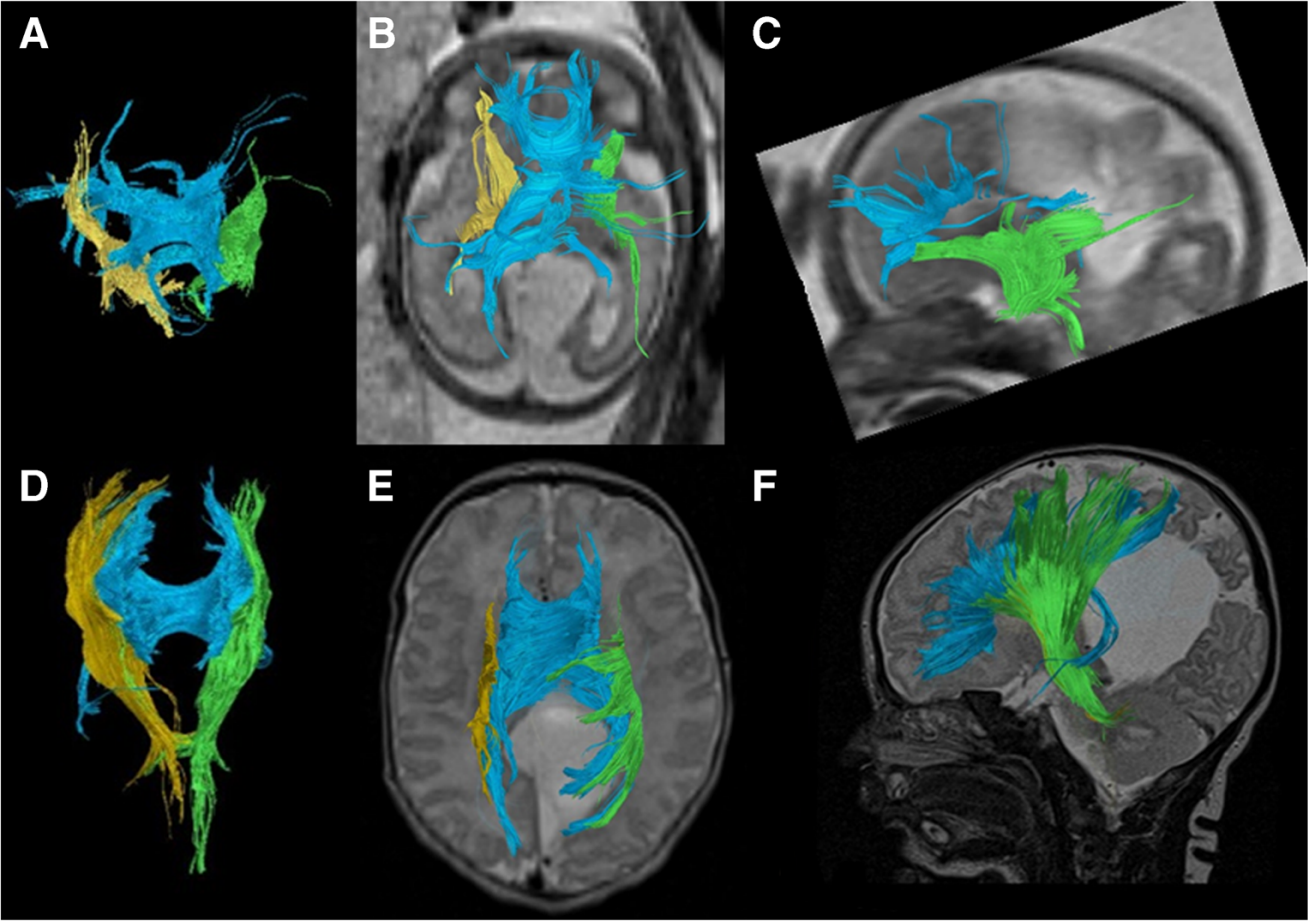
Fetal and postnatal corpus callosum and corticospinal tract segmentation in a boy (case 9) with a midline parieto-occipital cyst. **a–c** Age 29+4 gestational weeks. **a** Coronal diffusion tensor imaging (DTI) of segmented right (*yellow*) and left (*green*) corticospinal tracts superimposed with the corpus callosum (*blue*) in the fetal brain. **b** Overlay of the segmented fiber tracts on an axial T2-weighted image. **c** Sagittal view of the left corticospinal tract (*green*) reveals a tract that appears intact. Partially viewed is the corpus callosum (*blue*) as well on this T2-weighted image of the fetal brain. **d–f** Age 5 days. **d** Coronal DTI image of segmented right (*yellow*) and left (*green*) corticospinal tracts superimposed with the corpus callosum (*blue*) in the postnatal brain. **e** Axial view of the segmented tracts on a T2-weighted image shows lateral displacement of the fiber tracts posteriorly from the midline cyst. **f** Overlay of the segmented left corticospinal (*green*) and corpus callosum (*blue*) tracts on a sagittal T2-weighted image of the postnatal brain

Figure 7 illustrates segmented right (yellow fiber tracts) and left (green fiber tracts) corticospinal tracts in a case with a midline parieto-occipital cyst. Both corticospinal tracts were visualized on fetal and postnatal tractography and appeared to be intact although displaced posteriorly by the large cyst. The blue fiber tracts represented the callosal fibers. The prenatal segmentation of both the right and left corticospinal tracts was accurate of the postnatal segmentations.

**Fig. 8:**
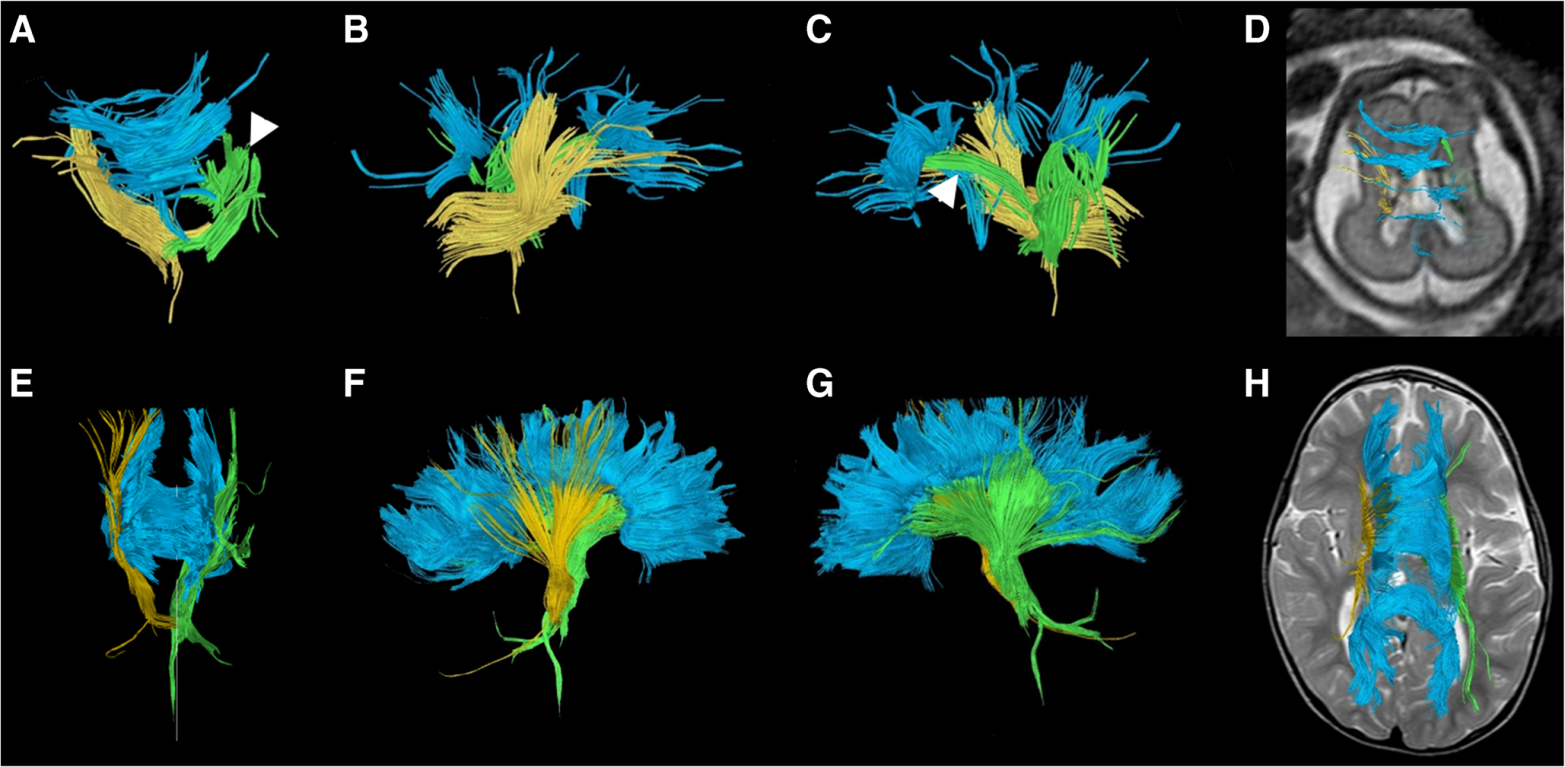
Fetal and postnatal diffusion tensor imaging corticospinal tract segmentation in a boy (case 2) with periventricular leukomalacia and cortical dysplasia. **a–d** Age 24+2 gestational weeks. **a** Coronal view of the segmented corpus callosum (*blue*) and bilateral corticospinal tracts (right, *yellow*; left, *green*). **b** Sagittal view of the segmented tracts shows the right corticospinal tract (*yellow*) to be present and intact. **c** Sagittal view of the segmented tracts shows the left corticospinal tract to appear discontinuous (*arrowhead*). **d** Overlay of the fiber tracts on an axial T2-weighted image for orientation. **e–h** Age 2 years. **e** Coronal view of the segmented corpus callosum (*blue*) and bilateral corticospinal tracts (right, *yellow*; left, *green*). **f** Sagittal view of the segmented tracts shows the right corticospinal tract (*yellow*) to be present and intact, although somewhat sparse. **g** Sagittal view of the segmented tracts shows the left corticospinal tract (*green*), appearing intact. **h** Overlay of all fiber tracts on an axial T2-weighted image for orientation

Figure 8 illustrates the corpus callosum and right and left corticospinal tracts in the fetal and 2-year-old images in a child with periventricular leukomalacia. The right corticospinal tract in the fetal segmentation appeared intact. However the left corticospinal tract appeared discontinuous. By contrast, the corticospinal tracts in the 2-year-old postnatal brain appeared intact bilaterally (Fig. 8). Noted was a slightly more sparse appearance of the right compared to the left corticospinal tract, which was thought to represent Wallerian degeneration as manifested by the severe appearance of white matter injury in the right cerebral hemisphere (Fig. 8).

**Fig. 9:**
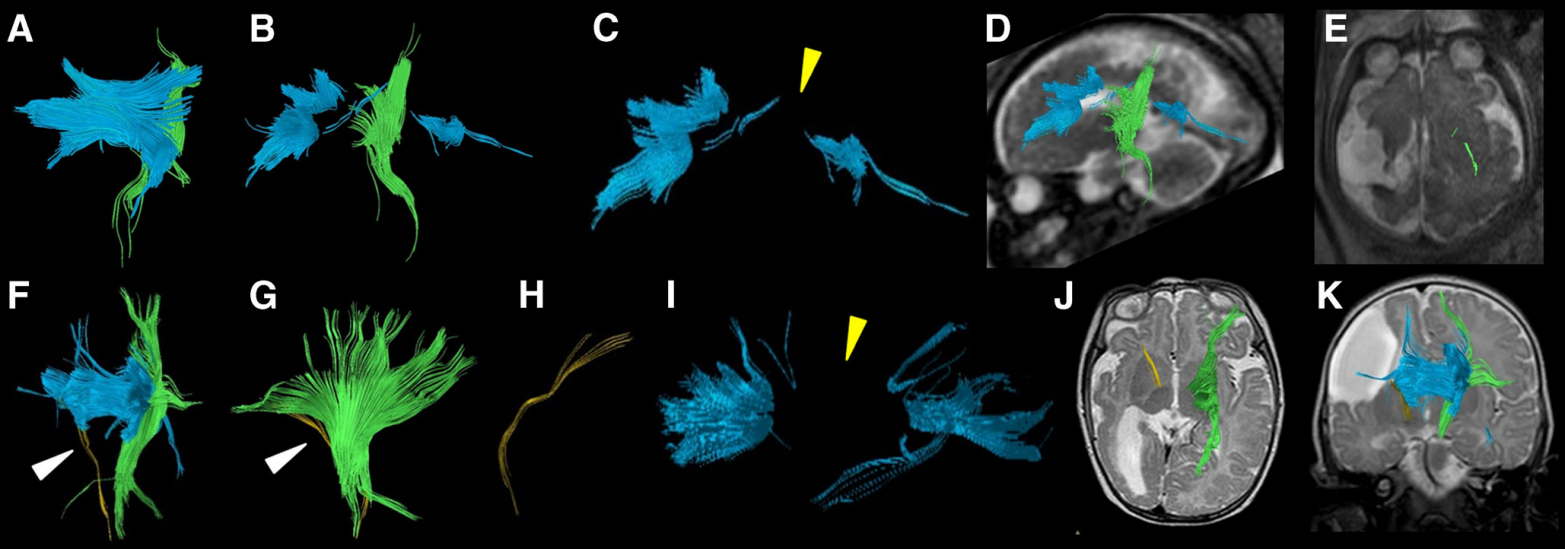
Fetal and postnatal diffusion tensor imaging corticospinal tract segmentation in a boy with right middle cerebral artery territory infarct (case 12). **a–e** Age 33+6 gestational weeks. **a** Coronal view of the segmentation shows the corpus callosum (*blue*) and left corticospinal tracts but not the right corticospinal tract. **b** Sagittal view shows the corpus callosum (*blue*) and left corticospinal tract (*green*). **c** Sagittal view of the corpus callosum reveals a paucity of tracts in the body segment of the corpus callosum (*yellow arrowhead*). **d** Overlay of the left corticospinal tract (*green*) and corpus callosum (*blue*) on a sagittal T2-weighted image is shown for orientation. **e** The left corticospinal tract traverses the expected region of the posterior third of the posterior limb of the internal capsule on axial T2-weighted MR image. **f–k** Age 4 months. **f** Coronal view of the segmentation shows the corpus callosum (*blue*), intact left corticospinal tract (*green*) and very few sparse fibers in the region of the right corticospinal tract (*arrowhead*), presumably virtually absent from significant right middle cerebral artery territory infarct. **g** Sagittal view of the intact left (*green*) and residual right (*arrowhead*) corticospinal tracts. **h** Sagittal view of the right corticospinal tract only (*yellow*). **i** Sagittal view of the corpus callosum reveals a paucity of tracts involving the body of the corpus callosum (*yellow arrowhead*), which is similar to the prenatal tractography (**c**). **j** Overlay of the segmented bilateral corticospinal tracts (*yellow*, right; *green*, left) on axial T2-weighted MR image for orientation shows the tracts traversing the expected region of the posterior third of the posterior limb of the internal capsule. **k** Overlay of the segmented bilateral corticospinal tracts (*yellow*, right; *green*, left) on coronal T2-weighted image for orientation

Figure 9 illustrates a case of a large right middle cerebral artery vascular territory infarct with ex vacuo dilatation of the right lateral ventricle. Fetal DTI tractography revealed no identifiable right corticospinal tract fibers, only left corticospinal tract fibers. In the postnatal DTI, a few residual fiber tracts composing the right corticospinal tract were noted, possibly reflecting atrophic or residual fiber tracts given their sparse appearance (Fig. 9). This appearance was markedly different from the left corticospinal tract in both the fetal and postnatal DTI segmentations. The prenatal segmentation of both the right and left corticospinal tracts was accurate with regard to the postnatal segmentations.

We analyzed whether a primary brain pathology versus secondary or unrelated (e.g., case 1, gastroschisis) brain abnormality decreased accuracy. Among cases with primary neurologic pathology (*n*=8), accuracy values were 88% (*n*=7 of 8) for the right corticospinal tract and 75% (*n*=6 of 8) for the left corticospinal tract. Positive predictive values were 100% for the right corticospinal tract and 83% for the left corticospinal tract. The NPV was 50% for the left corticospinal tract. No true-negative case emerged for the right corticospinal tract and an NPV could not be calculated. Among cases with secondary or unrelated pathology, 100% (*n*=4 of 4) of the cases revealed accurate results between fetal and postnatal segmentations for the right and 75% (*n*=3 of 4) for the left corticospinal tracts. Positive predictive values were 75% for the right and 100% for the left corticospinal tracts. Negative predictive value was 100% for the right corticospinal tract. No true-negative case among the four cases with secondary or unrelated pathologies was present for the left corticospinal tract and an NPV could not be calculated.

We also performed a subgroup analysis by fetal age (younger or older than 28 gestational weeks) for the corticospinal tracts. Among the fetuses younger than 28 gestational weeks, 100% (*n*=4 of 4) showed accurate results for the right and 75% (*n*=3 of 4) for the left corticospinal tracts. The PPVs were 100% for both the right and left corticospinal tracts. No true-negative case emerged and an NPV could not be calculated for either the right or left corticospinal tract. Among fetuses older than 28 gestational weeks, 88% (*n*=7 of 8) showed accurate results for the right and 75% (*n*=6 of 8) for the left corticospinal tracts. The PPVs were 100% for the right and 83% for the left corticospinal tracts. The NPVs were 50% for both the right and left corticospinal tracts.

**Fig. 10:**
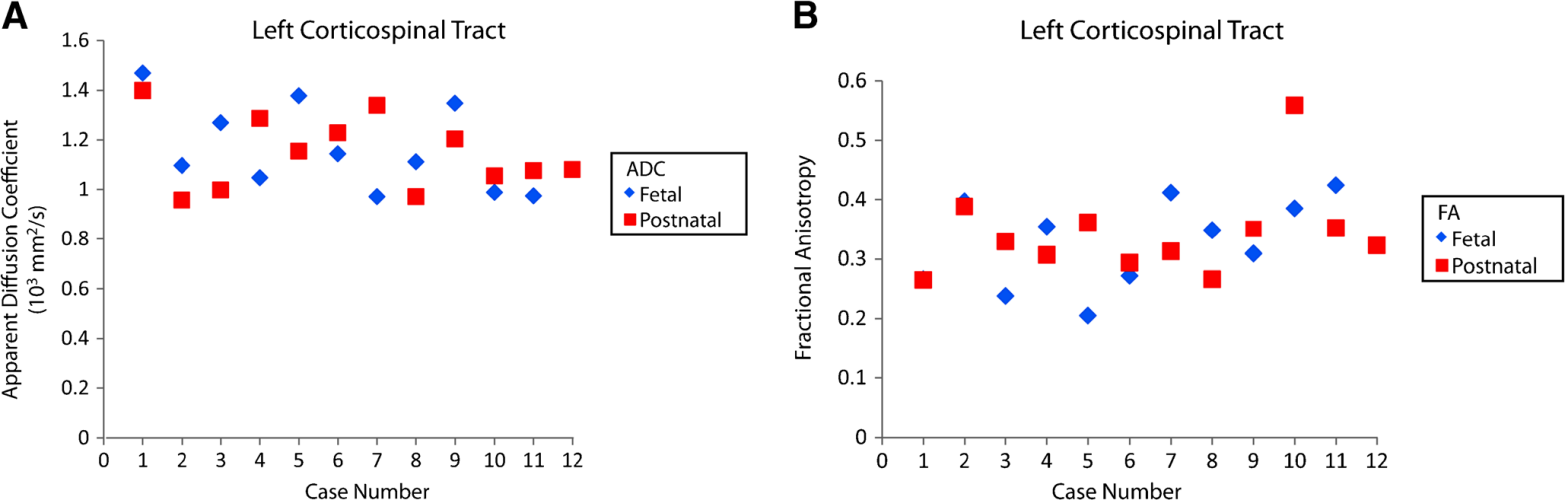
Fetal and postnatal left corticospinal tract apparent diffusion coefficient (ADC) and fractional anisotropy (FA). **a** Plot shows fetal and postnatal apparent diffusion coefficients for the left corticospinal tract by case. **b** Plot shows fetal and postnatal fractional anisotropy for the left corticospinal tract by case

**Fig. 11:**
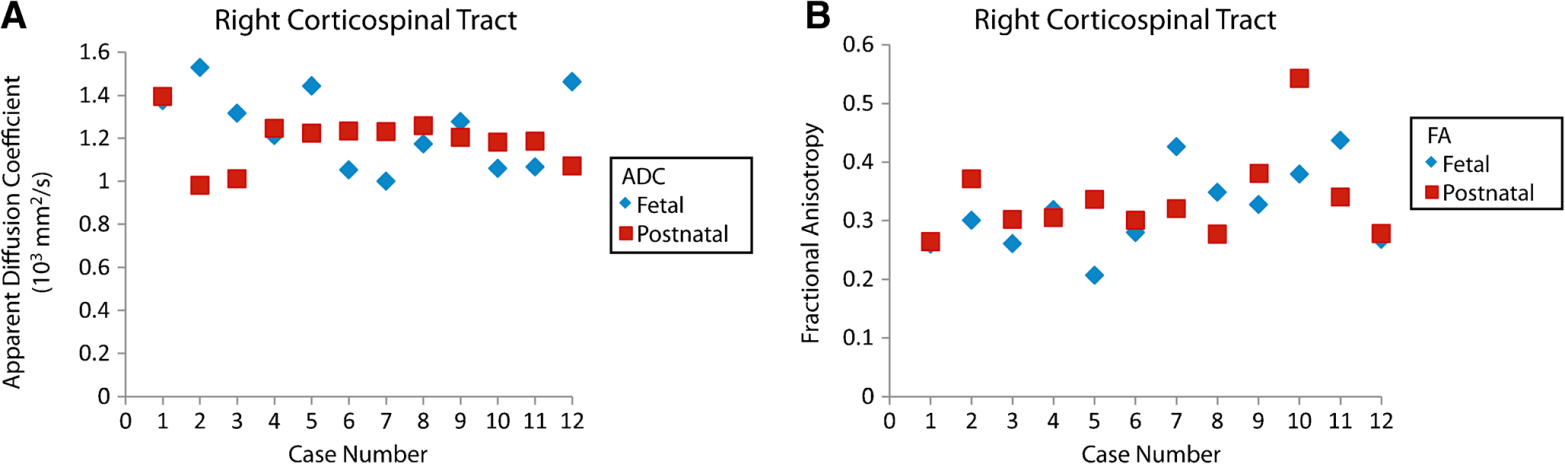
Fetal and postnatal right corticospinal tract apparent diffusion coefficient (ADC) and fractional anisotropy (FA). **a** Plot shows fetal and postnatal apparent diffusion coefficients for the right corticospinal tract by case. **b** Plot shows fetal and postnatal fractional anisotropy for the right corticospinal tract by case

Apparent diffusion coefficient and fractional anisotropy (FA) were visually graphed by paired cases. For the left corticospinal tract, the paired prenatal and postnatal cases revealed similar ADC and FA outcomes for the right and left corticospinal tracts (Figs. 10 and 11).

## Discussion

Many studies have examined individual fetal brains in vivo at a single time point [10, 15, 16]. However this study is the first to evaluate the fetal brain and subsequent postnatal brain from the same subject to assess the accuracy of the integrity of the fetal corpus callosum and corticospinal tracts. This unique dataset allows us to evaluate the accuracy of the tracts segmented from fetal MR examinations, particularly in a cohort of subjects with underlying pathology. Our results suggest there is a moderate to strong degree of accuracy and very high positive predictive values that range up to 100% in the characterization of these tracts by fetal DTI. These results indicate that when the corpus callosum and corticospinal tracts are visualized by tractography on fetal DTI, clinicians can have a high degree of confidence that these pathways will also be present postnatally.

### Corpus callosum

The corpus callosum, the largest commissural fiber tract, connects the two cerebral hemispheres. Studies have shown that dysgenesis or agenesis of the corpus callosum alters the structural connectome of the developing brain [4, 17]. Thus early detection of abnormal development of the corpus callosum is important to recognize during pregnancy because it could herald other underlying developmental abnormalities. Prenatal tractography results of the corpus callosum were accurate in more than two-thirds of the examined paired cases (Table 4), with accuracy values ranging between 67% and 75% and PPVs ranging between 50% and 100% for the four segments of the corpus callosum. These results suggest that if the corticospinal tract fiber pathways are visualized on prenatal DTI exams with a very high degree of confidence, the corticospinal tracts are likely to also be visualized on postnatal exams.

Interestingly we found poor visualization of the body of the corpus callosum, not only in prenatal but also postnatal tractography exams. This structure showed the most significant inconsistencies with structural imaging, where it could be visualized. The body of the corpus callosum is the thinnest segment of the corpus callosum, with a thickness in fetal brains at 24 gestational weeks of approximately 1.9 mm (range: 1.3–2.5 mm for the 5th–95th percentiles) as assessed by fetal ultrasound [18]. Also postnatally (0–2 months), the thickness of the mid-body of the corpus callosum has been described to be approximately 2.3 mm [19]. One study evaluated 152 neonates by ultrasound from ages 28 gestational weeks to 41 gestational weeks and reported no significant change in the thickness of the body of the corpus callosum, which measured approximately 2.7±0.4 mm [20]. In our study, with slice thicknesses of 3–4 mm for fetal DTI and 2–4 mm on postnatal DTI on 1.5-T MRI, diffusion information might have been lost because of partial volume effects in that specific region. Moreover, in four of the cases the corpus callosum was further thinned by hydrocephalus or ventriculomegaly, limiting the success of DTI in visualizing this segment. Notably, the body of the corpus callosum was not visualized in all four of these cases, yielding an accuracy of 100% in paired fetal and postnatal DTI tractography in cases with this structural morphology. These values contrasted with lower accuracy (63%) in cases without ventriculomegaly. It is, however, also important to note that in one case the body of the corpus callosum appeared to be absent on postnatal imaging, indicating that the absence of fiber pathways by tractography of the body of the corpus callosum in both the fetal and postnatal imaging might in some cases be true developmental absence of this structure (Fig. 2).

Interestingly, in two cases segments of the corpus callosum that were visualized by fetal tractography were not visualized in the postnatal exams (cases 8 and 11). Case 8 had a left middle cerebral artery infarct in the setting of tetralogy of Fallot; the body of the corpus callosum was visualized on fetal DTI but not on the postnatal DTI. Case 11 presented with septo-optic dysplasia and aqueductal stenosis; the splenium was visualized on fetal tractography but not on postnatal tractography. Explanations in these instances might be related to evolving changes of the original pathology (e.g., infarct or hydrocephalus) during development, sequelae of which might manifest more distinctly with maturity and development. Thus for certain types of injuries that continue to evolve during development, it is important to recognize that the fiber tracts might be restructuring as well.

### Corticospinal tracts

The corticospinal tracts revealed accuracies of 75–92% between paired fetal and postnatal tractography results. Similarly, the PPVs were 100% for the right and 88.9% for the left corticospinal tracts. These results suggest that if fiber pathways of the corticospinal tracts are visualized on prenatal DTI exams with a very high degree of confidence, the corticospinal tracts are also likely to be visualized on postnatal exams. The higher degree of accuracy of the corticospinal tracts compared to the corpus callosum could be related to a more advanced and mature stage of the fibers. Corticospinal tracts begin myelination at about 34 gestational weeks, whereas studies have suggested that callosal fibers do not myelinate until late third trimester [21–23].

Subgroup analyses assessing the type of injury (e.g., primary versus secondary/other, as defined in the methods section) did not reveal major differences in the accuracy of prenatal and postnatal tractography. Similarly, age (older or younger than 28 gestational weeks) did not appear to influence the results. However a larger sample size might be needed to better evaluate these potential confounders.

### Limitations

**Fig. 12:**
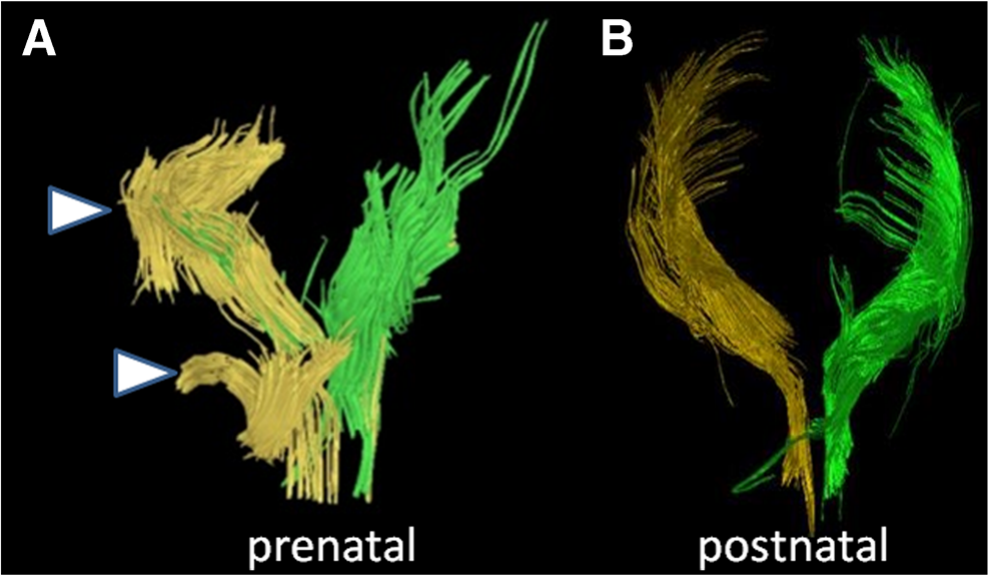
Inaccurate prenatal and postnatal tractography of the corticospinal tracts. **a, b** Case 4. **a** Prenatal (30+5 gestational weeks) and (**b**) postnatal (7 months) tractography pair of the corticospinal tracts are illustrated (*yellow*, right; *green*, left). Note the disrupted appearance of the fibers (*arrowheads* in **a**) at the proximal and distal aspects of the right corticospinal tract. This was thought to be a result of poor signal-to-noise ratio on the MRI. By contrast, the postnatal corticospinal tracts appear intact. This prenatal case was categorized as not being accurate for the right corticospinal tract because of the disrupted morphology

**Fig. 13:**
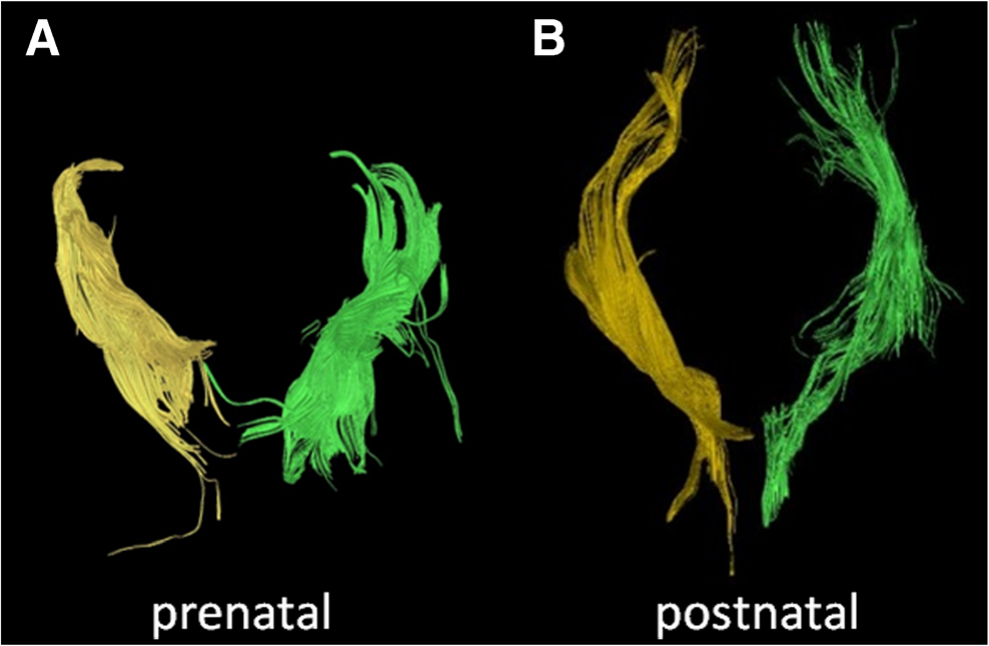
Accurate prenatal and postnatal tractography of the corticospinal tracts. **a, b** Case 11. The right and left corticospinal tracts of a (**a**) prenatal (28+2 gestational weeks) and (**b**) postnatal (2 days) tractography pair are shown. In this case, the prenatal tractography was considered accurate for the postnatal tractography appearance of the right and left corticospinal tracts

There are several limitations to this study. First, a few examinations had poor signal-to-noise ratio (SNR), which might have been minimized by using a 3-T MRI (Figs. 12 and 13). Although fetal MRI exams are presently routinely performed on a 1.5-T MRI, 3-T MRI can increase SNR, decrease acquisition time and increase spatial resolution [24, 25]. Motion degradation is another commonly encountered artifact in fetal MR imaging. The mothers are not sedated for the MR examination, and there are few other methods of minimizing fetal motion apart from altering the parameters to obtain faster acquisitions. Naturally, there are limitations to shortening acquisition times from the trade-off of SNR or spatial resolution.

Because of the retrospective nature of the study and the clinical indications of the respective exams, we included postnatal follow-up data from two 1.5-T scanners using standard neonatal DTI scanning parameters and acquisition times of about 4–6 min. However in comparing the data from both scanners there was no visible difference in the tractography results of the corpus callosum and corticospinal tracts.

A third limitation is the small sample size. Only 12 subjects met the inclusion criteria for the study with both fetal and follow-up postnatal MR imaging with DTI sequences. The high dropout rates can be explained by the fact that only cases with prenatally suspected brain abnormalities underwent postnatal MR imaging. At our institution early postnatal MRI is not routinely performed in cases without postnatal neurological deficits and with minor or normal CNS findings. Moreover, in cases with a poor neurodevelopmental prognosis, parents frequently opt for termination of pregnancy.

Another limitation is the variation in length of the interval time between fetal and postnatal imaging among the cases. The interval time ranged from 12 to 1,081 days. It is conceivable that cases with longer interval periods between the fetal and postnatal imaging and with earlier fetal ages might show in different results. The earliest fetal age was 23 gestational weeks, at which point the studied white matter tracts have developed. Nevertheless changes in the configuration of the white matter pathway and ongoing axonal growth certainly have influenced the appearance of the examined tracts. In order to detect and quantify these developmental changes, the inclusion of more cases and a study design using an atlas-based approach is required.

Last, given the 9-year span of the data collection, it is conceivable that the imaging parameters were optimized over time. Hence, a DTI examination from 2006 might have been of different quality from an examination performed in 2015. However, different parameters and coils were used for the fetal and postnatal DTI examinations, so we think this aspect of the retrospective study is noncontributory given the paired comparisons were already imaged differently. Moreover, in an attempt to objectively segment the fiber tracts two blinded investigators performed the segmentation on the corpus callosum and corticospinal tracts for each pair.

## Conclusion

Accounting for brain maturation, the results of this study indicate that prenatal visualization of the main projection and commissural tracts by DTI can be used as a positive predictive tool in the assessment of normal and pathological fetal brain development. Diffusion tensor imaging and tractography reveal important information about white matter pathways that no other prenatal imaging modality is able to provide. Here, we demonstrate that information about the presence and morphology of callosal or corticospinal pathways, as demonstrated by prenatal deterministic tractography, is positively predictive for the postnatal DTI results in a majority of cases. This holds true for abnormalities of these tracts as well as for their normal anatomy. Our data further support the concept of investing additional imaging time of up to 2 min to acquire DTI information, which allows for a more complete assessment of fetal brain malformations. Of note, we did encounter a low negative predictive value of our prenatal tractography results. Thus interpretation of “absent” white matter pathways should be undertaken with great care. However, further technical improvements (3 T, multichannel coils, faster acquisitions) might positively impact this limitation.

After including the postnatal neurological assessment the prospective clinical relevance of this advanced in utero neuroimaging technique can be fully appreciated. Detection of brain abnormalities alone is insufficient to provide in-depth counseling to parents. This emphasizes the need for further investigations of promising MR techniques such as DTI and tractography and the additional insight and hence impact on the possibility of prenatally detecting brain malformations.
